# 31-Year-Old Female Shows Marked Improvement in Depression, Agitation, and Panic Attacks after Genetic Testing Was Used to Inform Treatment

**DOI:** 10.1155/2014/842349

**Published:** 2014-03-18

**Authors:** Scott Lawrence

**Affiliations:** Centerstone, 1080 Ala Napunani Street No. 120, Honolulu, HI 96818, USA

## Abstract

This case describes a 31-year-old female Caucasian patient with complaints of ongoing depression, agitation, and severe panic attacks. The patient was untreated until a recent unsuccessful trial of citalopram followed by venlafaxine which produced a partial response. Genetic testing was performed to assist in treatment decisions and revealed the patient to be heterozygous for polymorphisms in *5HT2C, ANK3*, and *MTHFR* and homozygous for a polymorphism in *SLC6A4* and the low activity (Met/Met) *COMT* allele. In response to genetic results and clinical presentation, venlafaxine was maintained and lamotrigine was added leading to remission of agitation and depression.

## 1. Introduction

Patient treatment has traditionally been applied in a “one-size fits all” fashion. Most prescriptions have been standardized to target a particular illness or symptom which allows for minimal interindividual variation in treatment response. This becomes critical when treating psychiatric illnesses, as many prescribed medications are not effective in certain individuals or can have serious adverse effects. Information gathered from genetic testing can allow clinicians to predict a patient's propensity for medication response and risk for adverse drug reactions. Utilizing this information, a treatment plan can be customized to the patient to improve treatment outcomes [1].

## 2. Case

The patient, a 31-year-old female Caucasian with a long history of depression starting in her early teens, presented to a psychiatric nurse practitioner (PNP) with a chief complaint of incessant crying and worsening agitation. She reported symptoms of severe anergia, slow thought processes, short-term memory problems, and forgetfulness, all of which impacted daily activities, but she denied sleep disturbances. She reported multiple crying spells daily and admitted ongoing isolative behaviors. She also reported agitation leading to daily panic attacks but displayed no manic symptoms or psychosis. Her depression began in her midteens with progressively worsening of symptoms since that time.

The patient's psychiatric symptoms were managed solely by her primary care physician (PCP) who referred her to a PNP several times over the course of a year before the patient finally consented to be seen by a specialist. Although no official diagnosis was made prior to this visit, after seeing the PNP, she was diagnosed with major depressive disorder, recurrent and moderate (ICD-9 296.32/ICD-10 F33.1), with no presenting comorbidities. She has had no previous outpatient or inpatient treatment and denied suicidal ideation (SI) or attempts. The patient reported no significant medical history. Her family history is significant for unknown psychiatric treatment in her paternal grandmother, aunt, nephew, and brother. These family members were all psychiatrically hospitalized at least once for depression and another brother was diagnosed and treated for anxiety. The patient has two children from her previous marriage and is currently single after her divorce at age 28. Her depression symptoms have led to discussions with her employer about poor performance.

The patient was initiated on citalopram 20 mg by her PCP in January 2012 which was administered for one month but was reported to be entirely ineffective. The patient reports no other medication history. More recently, in March 2012, the patient was initiated on venlafaxine XR by the PNP and, after titration to 300 mg, produced a reduction in crying spells, depression, anxiety, and irritability. However, her symptoms still remained problematic and complaints of anergia and cognitive deficits were ongoing. Genetic testing was performed using the Genecept Assay in May 2012 to further guide treatment decisions. The Genecept Assay, designed by Genomind, LLC (Chalfont, PA), is a saliva based test which analyzes variations in ten genes associated with varied treatment response, treatment efficacy, side effect risk, and drug metabolism. The genes tested are summarized in Table 1. Genetic testing revealed the patient to be heterozygous for the variants rs3813929 of* 5HT2C*, rs10994336 of* ANK3*, and rs1801133 of* MTHFR* and homozygous for the variants rs63749047 of* SLC6A4* and low activity allele of* COMT*.

## 3. Discussion

Genotyping for* SLC6A4* revealed the patient to be at an increased risk for failure and/or intolerability with SSRI medications. Serotonin is removed from the synapse and returned to presynaptic cells by the serotonin transporter protein [2]. Variants in this gene result in altered transcription and consequently reduced levels of serotonin reuptake [3]. Several large meta-analyses have shown that the S allele correlates with slow response, poor response, and greater risk of side effects to selective serotonin reuptake inhibitors (SSRIs) [4, 5]. The mechanism of action of an SSRI selectively targets the serotonin transporter protein [6], and the presence of this variation may provide a potential explanation for the patient's failure with the SSRI citalopram. The patient's SSRI treatment history is limited, however, making it difficult to identify the exact cause of citalopram failure. Pharmacological agents that do not primarily target the serotonin transporter protein may be advantageous in patients who display this variation. Although the SLC6A4 variation may impact the serotonin pathway activity of venlafaxine, a commonly prescribed SNRI, the patient's partial response to this agent may be explained by its additional effects on the norepinephrine pathway. The decision to maintain venlafaxine was made with the patient reporting continued improvements in depression, anxiety, and irritability, as well as reductions in fatigue, better motivation, and some improvement to concentration and focus. Her children also noted that she seemed to be happier.

*5HT2C* is a site of antagonism by various neuroleptics. Serotonin signals satiety through this receptor [7]. Antagonism of 5HT2C has been shown to lead to increased food intake, hyperlipidemia, glucose intolerance, and obesity [8, 9]. The C allele of the-759C/T polymorphism confers risk for weight gain and metabolic syndrome, while the T allele exhibits protective effects for weight gain in patients taking atypical antipsychotics [9–11]. This patient is heterozygous for the C (high risk) allele and if atypical antipsychotics were a chosen intervention, additional vigilance may be appropriate to avoid and monitor weight gain.

The* ANK3* gene encodes for a protein crucial to the function of sodium ion channels, and variations in this gene may impact sodium channel activity [12]. The role of ANK3 in the brain includes the mediation of action potential firing and propagation and the modulation of neuronal excitability [12]. Altered ANK3 function could result in disruption of the proper development and function of neural circuits in the brain which regulate mood [12]. There have been numerous large genome-wide association studies that have found single nucleotide polymorphisms (SNPs) in* ANK3*, including rs10994336, to be correlated with, although not diagnostic for, bipolar disorder, cyclothymic mood disorders, and schizophrenia [13, 14]. SNPs in ANK3 have also been found to be associated with the predisposition of anhedonia, altered novelty seeking, impaired threat and stress signal processing, poorer cognition, and reduced integrity of white matter tracts [12]. The SNP, rs10994336, has not been demonstrated to have a direct impact on gene function; however, it serves as a true marker for another SNP, likely located nearby, which contributes to functional and structural changes in the brain related to symptomatology and risk for bipolar disorder [12]. Mood stabilizing agents, such as lamotrigine, which reduce neuronal excitability, may potentially be beneficial in patients with ion channel variations. Lamotrigine may affect the regulation of neurotransmission and action potential firing via the modulation of ion channel functioning [15]. Lamotrigine has long been used to successfully treat symptoms typically associated with bipolar disorder [15]. Lamotrigine was added as a therapeutic option to combat the patient's cognitive deficits and ongoing agitation and stress; it was chosen for its mechanism involving sodium channels as a potential modifier of the patient's ANK3 variation.

*COMT* is an enzyme responsible for breakdown of dopamine in the frontal lobes of the brain. Dopamine levels in this brain region are critical for memory, attention, judgment, and other executive functions [16, 17]. The literature suggests that this low activity variant may result in reduced dopamine degradation [18] leading to a hyperdopaminergic state and altered stress response [19]. As the patient's depression symptoms were improved with venlafaxine and lamotrigine was chosen to target the remaining symptoms, no treatments were chosen to target this variation at this time.

Methylfolate is the metabolically active form of folic acid and is essential in the catalytic reactions that produce the monoamine neurotransmitters norepinephrine, dopamine, and serotonin. Methylfolate is formed from folic acid through enzymatic conversion via MTHFR [20]. The risk allele of* MTHFR* leads to reduced thermodynamic stability and enzymatic activity [21, 22]. Augmentation with L-methylfolate has been demonstrated to produce benefits in several preliminary studies for patients with major depressive disorder [23]. As this patient was found to carry a risk allele in* MTHFR*, L-methylfolate 15 mg was initiated in June 2012 but was discontinued after one month due to fractional symptom reduction and a high copay cost.

## 4. Outcome 

After the addition of lamotrigine to the patient's treatment regime, the patient reported continued improvements in depression, anxiety, and mood lability. Low energy, cognitive impairments, and panic attacks also resolved. Additionally, although she had not previously admitted to paranoia in her clinical interviews, she reports that paranoia improved as well. The patient's response to the new medications was discussed at a follow-up appointment where she indicated a slight return of irritability. Lamotrigine was increased from 100 mg once a day in the morning (qam) to 200 mg qam to target the irritability. The depression and anxiety symptoms were well managed and the venlafaxine dosage was maintained at 300 mg qam. At the most recent follow-up appointment, the patient reported that all symptoms have resolved and she is doing well at her job with no more complaints about her performance. The patient self-initiated a medication washout period against clinical advice to determine if symptomology had resolved on its own, and within two weeks, symptoms had returned. She has since restarted these medications and has been stable with symptoms remaining in remission since returning to the previously achieved doses. Genetic testing proved to be a valuable clinical tool for this patient, guiding medication choices that dramatically impacted underlying symptomatology.

## Figures and Tables

**Table 1:** Genes tested using the Genecept Assay.

Gene	Variant	Functional significance
Serotonin transporter (SLC6A4)	Long/short (rs63749047)	Reduced serotonin reuptake
	A > G (rs25531)	
Serotonin receptor subtype 2C (5HT2C)	−759 C > T (rs3813929)	Altered satiety signaling
Dopamine receptor subtype 2 (DRD2)	141 C INS/DEL (rs1799732)	Altered binding of dopamine and antipsychotics
Voltage-dependent calcium channel L-type, alpha 1c subunit (CACNA1C)	G > A (rs1006737)	Altered neuronal depolarization
Ankyrin G (ANK3)	C > T (rs10994336)	Dysregulation of sodium channels
Catechol-O-methyltransferase (COMT)	158 Val > Met (rs4680)	Altered dopamine degradation
Methylenetetrahydrofolate reductase (MTHFR)	677 C > T (rs1801133)	Impaired folic acid metabolism
Cytochrome P450 2D6 (CYP2D6)	Multiple variations	Variants can lead to poor metabolism, intermediate metabolism, or ultra- metabolism of certain medications
Cytochrome P450 2C19 (CYP2C19)	Multiple variations	Variants can lead to poor metabolism, intermediate metabolism, or ultra- metabolism of certain medications
Cytochrome P450 3A4/5 (CYP3A4/5)	Multiple variations	Increased metabolism of certain medications
